# Short-Cycle Therapy with Bictegravir/Emtricitabine/Tenofovir Alafenamide in a Small Cohort of Virally Suppressed People Living with HIV: A Long-Term Follow-Up

**DOI:** 10.3390/biomedicines12112620

**Published:** 2024-11-15

**Authors:** Massimiliano Lanzafame, Emanuela Lattuada, Andrea Delama, Giovanni Mori, Sandro Vento

**Affiliations:** 1 Unit of Infectious Diseases, Santa Chiara Hospital, Azienda Provinciale per i Servizi Sanitari, 38122 Trento, Italy; emanuela.lattuada@apss.tn.it (E.L.); giovanni.mori@apss.tn.it (G.M.); 2 Faculty of Medicine, University of Puthisastra, Phnom Penh 12211, Cambodia; ventosandro@yahoo.it

**Keywords:** antiretroviral treatment, short-cycle therapy, HIV, virologically suppressed people, bictegravir

## Abstract

Background: Antiretroviral triple therapy has considerably reduced morbidity and mortality in people living with HIV and is the standard-of-care treatment. However, it is lifelong and linked to long-term side effects and adherence problems. Methods: Here, we report long-term virological and immunological outcome in 12 virally suppressed people on short-cycle therapy with bictegravir/emtricitabine/tenofovir alafenamide administered five days a week (Monday to Friday). Results: All patients, after a long term follow-up, were virally suppressed Conclusions: In the wait for new long-acting antiretroviral drugs and new antiretroviral formulations, short-cycle therapy has proven to be a safe and effective alternative to the standard daily antiretroviral regimen for individuals living with HIV who are virologically suppressed.

## 1. Introduction

Antiretroviral triple therapy has dramatically reduced morbidity and mortality in people living with HIV (PLHIV), becoming the standard-of-care treatment. While advances in treatment have greatly improved the life expectancy of PLHIV, some critical aspects remain. Among these, the medium- to long-term side effects of taking antiretroviral drugs and the large number of pharmacologically active molecules to be taken daily are the two main factors affecting patient compliance; adequate adherence is essential to ensure therapeutic success. The chronicity of HIV infection, moreover, has exposed PLHIV to a series of previously unconsidered comorbidities typical of ageing.

Within this scenario, research is moving towards optimising therapeutic management by reducing the daily and cumulative antiretroviral drug burden over time (pill burden) and limiting drug interactions with other drugs taken by the patient concomitantly. Optimization is especially important for the frail and/or elderly PLHIV who have an inflammatory state due to the chronic infection and multiple comorbidities that need to be treated, with the consequent risk of exposure to drug interactions. The above, coupled with the possible appearance of long-term toxicity of antiretroviral drugs, can cause a risk of “prescription cascades”.

In recent years, various strategies have been investigated to reduce antiretroviral drug exposure for PLHIV and enhance their adherence and quality of life while ensuring safety and maintaining virological suppression. Examples of these strategies are dual therapies (for instance, dolutegravir and lamivudine or rilpivirine) and the recent introduction in clinical practice of long-acting injectable regimens (rilpivirine and cabotegravir). In the not-too-distant future, ultra-long-acting regimens, antiretroviral nano-formulations, microarray patches, and broadly neutralizing antibodies will be available [1]. Meanwhile, the “intermittent” or “short-cycle” therapy (SCT) has gathered significant attention, particularly in France [2]. This approach allows individuals living with HIV to take their antiretroviral medication at standard doses and in combination for a limited number of consecutive days each week (typically four or five), followed by a break during the weekend (from Friday to Sunday or Saturday to Sunday). This strategy has employed non-nucleoside reverse transcriptase inhibitors, integrase strand transfer inhibitors, and boosted protease inhibitors [3]. Multiple clinical studies and trials have assessed SCT, with regimens varying in duration (e.g., 5-days-on/2-days-off, 4-days-on/3-days-off) [4].

Integrase strand transfer inhibitors (INSTIs), due to their favorable forgiveness [5], are preferred. Dolutegravir and bictegravir dissociate slowly from the INSTIs-DNA complex, and their half-lives are approximately 12 h and 17 h, respectively, and thus longer than raltegravir and elvitegravir [6]. Thus, dolutegravir and bictegravir are the favored antiretroviral drugs for intermittent dosing.

Here, we report the long-term virological outcome in 12 virally suppressed patients on short-cycle therapy with bictegravir/emtricitabine/tenofovir alafenamide, administered five days a week (Monday to Friday).

## 2. Case Series

In the Infectious Diseases Unit of Santa Chiara Hospital in Trento (Northern Italy), we regularly follow 629 PLHIV. Among these, at the beginning of March 2022, twelve virologically suppressed individuals (three women) on a stable bictegravir/emtricitabine/tenofovir alafenamide antiretroviral daily regimen started a “short-cycle” (Monday to Friday) therapy. All these people were selected after reporting, during their regular monitoring visits, that they often skipped, in the six previous months, a few doses of the drug compared to others (79 patients) who were taking bictegravir/emtricitabine/tenofovir alafenamide regularly. All the individuals signed an informed consent form, as required by the “off-label” use of drugs in Italy. In Table 1, people’s characteristics at baseline are reported. The mean age was 55.9 years (range: 25–75). The mean antiretroviral therapy duration was 18 years (range 4–32), the mean duration of virological suppression was 12.5 years (range: 4–25), and the mean time from the diagnosis of HIV infection was 20 years (range: 4–33) before the beginning of short-cycle therapy. Most people had been on various antiretroviral regimens (mean number: 6; range: 1–13); however, they had not experienced any virological failure after starting their first regimen, and the mean CD4+ lymphocyte count nadir was 373.9/µL (range 15–998). All patients had plasma HIV RNA < 20 copies/mL at the beginning of the short-cycle therapy. Plasma HIV RNA was quantified one and three months after the switch, and every six months thereafter; blood samples were collected on Monday (more than 48 h after stopping bictegravir/emtricitabine/tenofovir alafenamide and before restarting it). At the time of writing (November 2024), all patients are still aviremic and have a mean follow-up of 27.8 months (range 27–31). The mean CD4+ve cell count rose from 683/µL (at the beginning of the short-cycle therapy) to 929/µL (at the last visit). The mean body weight was 72.1 Kg at the time of switch and 71.9 Kg at the last control. No patients developed metabolic and/or cardiovascular diseases or opportunistic infections during the short-cycle therapy.

## 3. Discussion

In our small cohort, all people, who were on a stable bictegravir/emtricitabine/tenofovir alafenamide antiretroviral daily regimen and in virological suppression, have maintained a plasma viral load of less than 20 copies/mL for over 24 months, and their CD4+ T cell count rose during follow-up.

Various studies have shown the virological safety of a short-cycle therapy, starting with the FOTO study in 2007 [7]. The BREATHER study (“BREaks in Adolescent and Child Therapy using Efavirenz and two NRTIs”), a multicenter, randomized, controlled, open-label phase 2/3 trial, confirmed the non-inferiority of a 5-day-per-week SCT compared to the standard 7-day-per-week regimen in 199 adolescents on efavirenz-based ART [8]. Subsequently, in the “Intermittent, in careful short-cycles, antiretrovirals may retain efficacy” (ICCARRE) study, virological suppression was reported in all patients on a 4-days-on/3-days-off scheme [9]. The use, in some cases, of a protease inhibitor as third agent, which has fairly short half-life, suggested that factors other than half-life played a role in maintaining virological suppression during the “off” period. Six other studies have focused on a 4-days-on/3-days-off antiretroviral strategy [3,10,11,12,13,14]. In 2009, Rudy and coworkers reported that viral rebounds occurred in 12 of 32 adolescents followed for 48 weeks [10]; these somehow-disappointing results were attributed both to the short half-life of protease inhibitors and to the fact that the adolescents had a long history of antiretroviral treatments, and a few of them had many resistance mutations.

More recently, the ANRS 170 QUATUOR trial, a randomized, open-label, multicenter, non-inferiority study, confirmed that the 4-days-on/3-days-off strategy was non-inferior in a large cohort of virologically suppressed PLHIV on various ART regimens over a 96-week follow-up period [3]. Small observational studies using, as a third agent, bictegravir, doravirine, or rilpivirine have confirmed the efficacy and safety of this strategy [14,15,16]. Some of the above studies have also reported the plasma concentrations of antiretrovirals [3,8,16]. In particular, in the ANRS 170 QUATUOR trial, the median concentrations of antiretroviral drugs were much lower in the intermittent arm than in the continuous treatment [3]. Sellem and colleagues evaluated 85 virally suppressed PLHIV switched to intermittent bictegravir/emtricitabine/tenofovir alafenamide treatment, administered 4 or 5 days a week [15]. Notwithstanding its limitations (i.e. its observational nature and the small number of people included), the study found a high level of virological success (100% at week 48 and 97.6% at week 96). Two people with poor adherence had a virological failure without the development of resistance mutations at HIV genotypic testing [15]. Virological re-suppression was obtained after resuming daily bictegravir/emtricitabine/tenofovir alafenamide treatment. The authors performed a pharmacokinetics analysis in a subset of individuals; in 22 out of 38, the C trough of bictegravir was below the protein-adjusted EC_95_ (162 ng/mL for the wild-type) [15].

Similarly, in 115 samples obtained after the 3-days-off treatment to determine the lowest quantifiable concentration, we observed that 71.3% of the plasma samples had rilpivirine concentrations below the efficacy threshold of 50 ng/mL, and 37.4% were below the 90% inhibitory concentration (IC90) of 12 ng/mL [14].

However, in all the above studies, the intracellular concentrations of antiretroviral drugs were not measured; hence, we cannot exclude the hypothesis of effective concentrations of the drugs inside PBMCs. Leibowitch and colleagues, through the ICCARRE project, proposed an explanation for the success of an intermittent strategy. They suggested that HIV rebound occurs only after an eclipse phase lasting from 1 to 7 days or even longer following the interruption of effective therapy. This phenomenon is particularly noticeable in individuals living with HIV who have a prolonged history of virological suppression, reduced viral loads, and a less-activated lymphoid system, which is less conducive to HIV replication. Under these circumstances, lower concentrations of antiretroviral drugs are still effective in controlling viral replication [9]. A study from 2010 further demonstrated that the longer the period of viral suppression, the more drug doses could be missed without leading to virological failure [17]. The reasoning behind this finding is that reduced drug exposure suffices to prevent virological failure because the overall viral burden diminishes over time [17]. From a pharmacological point of view, bictegravir seems the most suitable INSTI for use in intermittent antiretroviral therapy. The forgiveness of bictegravir and of other INSTI-containing regimens has been evaluated in vivo [18] and In Vitro [19]. Maggiolo and colleagues, in a retrospective study including 281 PLHIV treated with bictegravir/emtricitabine/tenofovir alafenamide, showed, through adherence measurement, an elevated rate of forgiveness associated with a high rate of virological suppression [18].

Acosta and colleagues studied In Vitro four INSTI-containing regimens (bictegravir/emtricitabine/tenofovir alafenamide, dolutegravir with emtricitabine/ tenofovir alafenamide, dolutegravir/lamivudine, and dolutegravir/rilpivirine) [19], evaluating the time to In Vitro virological breakthrough and the emergence of resistance mutations (resistance barrier) at full or suboptimal treatment adherence levels. Bictegravir/emtricitabine/tenofovir alafenamide, when suboptimal treatment adherence level was tested, was the combination with the highest forgiveness and barrier to resistance [19].

In the same study, dual therapy (dolutegravir/lamivudine and dolutegravir/rilpivirine combinations) was weaker, in terms of viral breakthrough and the emergence of resistance mutations, than triple antiretroviral combinations for drug concentrations corresponding to having missed two or more consecutive doses [19].

Recently, a randomized, open-label, multicentric study (“ANRS 177 DUETTO”) evaluating a two-drug antiretroviral therapy (65.6% of participants were on a dolutegravir/lamivudine combination) taken four days a week rather than daily, found a similar rate of suppression but higher rates of treatment failure in the intermittent arm [20]. Six patients out of eight on virological failure were on dolutegravir/lamivudine; interestingly, only two of the eight patients had a low plasma drug concentration.

Future research should focus on understanding how intracellular concentrations of antiretrovirals, particularly within peripheral blood mononuclear cells (PBMCs), impact viral suppression. Studies could also investigate pharmacokinetics under different SCT patterns (e.g., 5-days-on/2-days-off vs. 4-days-on/3-days-off) to optimize dose regimens and improve SCT’s efficacy across diverse patient profiles. Individual differences in immune responses, HIV reservoir sizes, and pharmacogenomics could play a role in SCT’s success. Tailoring SCT to patient-specific factors could improve outcomes, especially for those with lower nadir CD4+ counts or those who experience periodic low-level viral blips. Another future research direction could be to study the efficacy of short-cycle therapy in patients with long-term viral suppression and stable health status. Investigating its efficacy and safety in older adults or those with comorbidities could clarify SCT’s broader applicability, especially in contexts where polypharmacy and drug interactions are concerns. SCT’s long-term impact on immune activation and inflammatory biomarkers remains underexplored; studies could examine whether SCT reduces chronic inflammation, which is often linked to HIV and its treatment, potentially improving long-term health outcomes. The identification of biomarkers that predict a patient’s response to SCT could aid in selecting candidates who are more likely to benefit from intermittent therapy. This would enhance SCT’s precision and potentially extend its use to a broader range of patients.

SCT could also significantly reduce ART expenses, allowing for broader access to treatment. By reducing the overall drug burden, SCT may ease the financial strain on health systems, particularly in resource-limited settings. Recently, the ANRS 170 QUATUOR study demonstrated that the “4/7-day” strategy’s cost-effectiveness enables savings amounting to one third of the total direct medical costs, while maintaining ART effectiveness, in PLHIV in France [21].

Thanks to the reduced dose frequency, SCT could improve adherence and lessen treatment fatigue, leading to better outcomes for PLHIV. This may be especially beneficial for patients who experience psychological or physical challenges with daily regimens. 

SCT’s promising results may inform updates in HIV treatment guidelines, particularly as a strategy for treatment optimization in stable, virologically suppressed individuals. Protocols could be developed to incorporate SCT as a potential standard of care for suitable candidates.

These directions and applications reflect SCT’s potential to revolutionize HIV treatment by reducing the drug burden, improving adherence, and enhancing the quality of life for individuals living with HIV. Further research could lead to more robust, cost-effective, and patient-friendly HIV management strategies.

## 4. Conclusions

Bictegravir/emtricitabine/tenofovir alafenamide seems the most appropriate combination for use in strategies of intermittent antiretroviral treatment due to its antiviral potency and forgiveness.

While waiting for new, potent, long-acting drug formulations and different means of delivery, oral short-cycle antiretroviral therapies represent a feasible and valuable option for treatment optimization in selected individuals. This strategy can help to improve patient quality of life, reducing pills fatigue and stigma, while also decreasing antiretroviral costs.

## Figures and Tables

**Table 1:** Baseline patients characteristics.

MEAN AGE (years)	55.9 (25–75)
CAUCASIAN	12
GENDER (male/female)	9/3
RISK GROUP (MSM, heterosexual, intravenous drug use)	7 MSM; 1 Heterosexual; 4 with a previous history of intravenous drug use
CDC STAGE	5 patients A1; 1 A3; 2 B2; 1 B3; 3 C3
MEAN CD4+ LYMPHOCYTE COUNT NADIR (no./µL)	373.9 (15–998)
MEAN WEIGHT AT SWITCH (Kg)	72.100 kg
MEAN WEIGHT AFTER MORE THAN 24 MONTHS OF “SCT”	71.900 kg
MEAN DURATION OF HIV INFECTION *	20 years (4–33)
MEAN DURATION OF ANTIRETROVIRAL THERAPY *	18 years (4–32)
MEAN DURATION OF VIROLOGICAL SUPPRESSION *	12.5 years (4–25)
MEAN NUMBER OF ANTIRETROVIRAL REGIMENS BEFORE BICTEGRAVIR/EMTRICITABINE/TENOFOVIR ALAFENAMIDE	6 (1–13)
MEAN LYMPHOCYTE CD4+ LYMPHOCYTE COUNT AT SWITCH (no./µL)	683/µL

* Before start of short-cycle therapy (5-days-on/2-days-off).

## Data Availability

Data supporting the reported results can be found in our archive database.
